# Promoting hygienic hand disinfection as an ongoing task: results of a cluster-randomized controlled trial to (re-)raise compliance of physicians and nurses based on psychological tailoring in a tertiary intensive care setting

**DOI:** 10.1186/2047-2994-4-S1-O47

**Published:** 2015-06-16

**Authors:** T von Lengerke, B Lutze, C Krauth, K Lange, JT Stahmeyer, IF Chaberny

**Affiliations:** 1 Medical Psychology Unit, Hannover Medical School, Hannover, Germany; 2 Institute of Hygiene/Hospital Epidemiology, Leipzig University Hospital, Leipzig, Germany; 3 Institute for Epidemiology, Social Medicine and Health Systems Research, Hannover Medical School, Hannover, Germany; 4 Institute for Medical Microbiology and Hospital Epidemiology, Hannover Medical School, Hannover, Germany

## Introduction

Insufficient use of psychological theory is one reason that conclusive evidence regarding hand hygiene promotion is scarce. In addition, compliance has been shown to be lower among physicians than among nurses. The PSYGIENE-project set out to draw on theoretical advances (Health Action Process Approach-HAPA) to optimise education and feedback interventions.

## Objectives

To test whether psychologically tailored interventions lead to higher increases of hand hygiene compliance than usual care (German Clean Care is Safer Care-campaign).

## Methods

A cluster-randomized controlled trial was conducted on intensive care and hematopoietic stem cell transplantation units of Hannover Medical School, a tertiary university hospital. Clusters were defined by classifying wards as early/late adopters by 2008-12 compliance. Tailoring targeted wards and was informed by problem-focused interviews with physicians and chief nurses (response rates: 100%) and a written survey which assessed HAPA-factors (physicians: 71%; nurses: 63%). The outcome was 2014 compliance observed by WHO-standards.

## Results

In 2013, 15 education sessions for physicians (participation rate: 46%) and 39 for nurses (50%) and 12 feedback meetings with chief nurses (100%) were conducted. Overall, from 2013-14 compliance increased from 48 to 63% (physicians) and 56 to 67% (nurses). Increases on the 6 tailored wards was not greater than given usual care (10 vs. 13%, p=0.126). This held both for physicians, among whom tailoring even led to a significantly lower increase than usual care among late adopter-wards (7 vs. 23%, p=0.046), and nurses (10 vs. 11%, p=0.590).

## Conclusion

Compliance increased both in the tailored and the usual care-group. While explanations of this result (e.g. study design issues or insufficiency of psychological theories of population behaviour to explain organisational behaviour) remain speculative, the overall increase in compliance does stress behavioural strategies to promote hand hygiene compliance as an ongoing task in which to continuously (re-)invest.

## Disclosure of interest

None declared.

